# MS4A1 Dysregulation in Asbestos-Related Lung Squamous Cell Carcinoma Is Due to CD20 Stromal Lymphocyte Expression

**DOI:** 10.1371/journal.pone.0034943

**Published:** 2012-04-13

**Authors:** Casey M. Wright, Santiyagu M. Savarimuthu Francis, Maxine E. Tan, Maria U. Martins, Clay Winterford, Morgan R. Davidson, Edwina E. Duhig, Belinda E. Clarke, Nicholas K. Hayward, Ian A. Yang, Rayleen V. Bowman, Kwun M. Fong

**Affiliations:** 1 School of Medicine, The University of Queensland Thoracic Research Centre, Queensland, Australia; 2 Department of Thoracic Medicine, The Prince Charles Hospital, Queensland, Australia; 3 Oncogenomics Laboratory, Queensland Institute of Medical Research, Queensland, Australia; 4 Department of Anatomical Pathology, The Prince Charles Hospital, Queensland, Australia; 5 Histotechnology Facility, Queensland Institute of Medical Research, Queensland, Australia; Cincinnati Children's Hospital Medical Center, United States of America

## Abstract

Asbestos-related lung cancer accounts for 4–12% of lung cancers worldwide. We have previously identified *ADAM28* as a putative oncogene involved in asbestos-related lung adenocarcinoma (ARLC-AC). We hypothesised that similarly gene expression profiling of asbestos-related lung squamous cell carcinomas (ARLC-SCC) may identify candidate oncogenes for ARLC-SCC. We undertook a microarray gene expression study in 56 subjects; 26 ARLC-SCC (defined as lung asbestos body (AB) counts >20AB/gram wet weight (gww) and 30 non-asbestos related lung squamous cell carcinoma (NARLC-SCC; no detectable lung asbestos bodies; 0AB/gww). Microarray and bioinformatics analysis identified six candidate genes differentially expressed between ARLC-SCC and NARLC-SCC based on statistical significance (*p*<0.001) and fold change (FC) of >2-fold. Two genes *MS4A1* and *CARD18*, were technically replicated by qRT-PCR and showed consistent directional changes. As we also found *MS4A1* to be overexpressed in ARLC-ACs, we selected this gene for biological validation in independent test sets (one internal, and one external dataset (2 primary tumor sets)). *MS4A1* RNA expression dysregulation was validated in the external dataset but not in our internal dataset, likely due to the small sample size in the test set as immunohistochemical (IHC) staining for *MS4A1* (CD20) showed that protein expression localized predominantly to stromal lymphocytes rather than tumor cells in ARLC-SCC. We conclude that differential expression of *MS4A1* in this comparative gene expression study of ARLC-SCC versus NARLC-SCC is a stromal signal of uncertain significance, and an example of the rationale for tumor cell enrichment in preparation for gene expression studies where the aim is to identify markers of particular tumor phenotypes. Finally, our study failed to identify any strong gene candidates whose expression serves as a marker of asbestos etiology. Future research is required to determine the role of stromal lymphocyte *MS4A1* dysregulation in pulmonary SCCs caused by asbestos.

## Introduction

Despite advances in research and treatment, lung cancer remains one of the leading causes of death globally, with five-year survival rates as low as 15% [1]. While the majority of lung cancers develop in smokers [2], other carcinogens including asbestos may contribute to lung cancer development [3]. The contribution of asbestos to lung cancer in persons exposed to both tobacco and asbestos is difficult to quantify because of interactions between the two agents in initiating and promoting neoplastic changes. Apart from a history of exposure, there are no clinicopathologic criteria distinguishing asbestos-related and tobacco-related lung cancers [4]. Recently, we and others have reported gene expression profiles that can potentially differentiate between these subtypes [5].

Although lung cancer histopathologic subtypes observed in persons with and without asbestos exposure are similar [6], [7], [8], evidence is accruing that primary adenocarcinomas and squamous cell carcinomas (SCC) of the lung arise by distinctly different carcinogenic pathways and display different sensitivities to targeted therapies. We recently identified *ADAM28* as a candidate oncogene in asbestos-related lung adenocarcinomas [9]. Here we compare gene expression between asbestos-related (ARLC-SCC) and non-asbestos related primary lung squamous cell carcinomas (NARLC-SCC). Our aims were 1) to determine whether SCC gene expression profiles differed between individuals with and without evidence of prior asbestos exposure as determined by pulmonary asbestos lung fiber count, and 2) to discover and validate candidate gene expression biomarkers of ARLC-SCC that could be potential diagnostic markers.

## Materials and Methods

### Ethics Statement

This study was approved by The Prince Charles Hospital Human Research Ethics Committee and subjects gave informed written consent at the time of surgical resection for donation of resected tissue for this research.

### Study Population

The study population consisted of a training set of 56 cases of primary lung SCC. Lung asbestos body count was measured in uninvolved ‘normal’ (non-tumor) lung tissue using the bleach digestion technique initially described by Roggli [10] and previously outlined [11]. Urban dwellers without occupational asbestos exposure have been found to have fewer than 20 asbestos bodies per gram wet weight (AB/gww) using this method [12]. SCC cases were classified as “asbestos exposed” (n = 26) if there were ≥20 AB/gww in their non-tumor tissue, or “non-exposed” (n = 30) if no asbestos bodies were found. We also used an internal test set consisting of 3 ARLC-SCC and 9 NARLC-SCC for biological validation. Subject demographics and tumor characteristics are listed in Table 1.

**Table 1:** Demographics for the TPCH Training and Independent Test sets.

	Training Set			Test Set		
	ARLC (>20AB/gww)	NARLC (0AB/gww)	*P-*value	ARLC (>20AB/gww)	NARLC (0AB/gww)	*P*-value
**N**	26	30		3	9	
**Age** (*Years, Mean ± 1 SD*)	65.8 (10.6)	66.4 (8.7)	0.812	67.0 (5.2)	67.4 (8.7)	0.943
**Sex**						
*Male*	20	24	0.78	3	7	0.371
*Female*	6	6		0	2	
**Smoking History**						
*Current*	5	13	0.110	1	3	0.827
*Former*	18	16		2	5	
*Never*	3	1		0	1	
*Pack years (Mean ± 1 SD)*	44.4 (51.1)	51.3 (23.8)	0.528	62.7 (32.5)	66.5 (51.5)	0.908
**TNM Tumor Stage**						
*(IA and IB)*	13	12	0.361	3	6	0.632
*(IIA and IIB)*	10	10		0	1	
*(IIIA and IIIB)*	3	8		0	2	
**Self-reported asbestos exposure**						
*Yes*	7	5	0.192	2	2	0.083
*No*	10	20		0	0	
*Unknown*	1	1		1	6	
*Missing*	8	4		0	1	
**Asbestos Fibre Count** *(Mean ± 1 SD)*	77.7 (103.5)	0 (0)	0.001	61.8 (48.0)	0 (0)	0.002

ARLC – Asbestos-related lung cancer; NARLC – Non-asbestos related lung cancer; AB/gww – asbestos bodies/gram wet weight; SD - standard deviation; AB – asbestos bodies.

### RNA Extraction and Gene Expression Analysis

Total RNA was extracted from 20–30 mg of fresh frozen lung tumor tissue using the TRIZol™ reagent (Invitrogen, CA, USA), cleaned using miRNeasy Kits (Qiagen, MD, USA) and reverse transcribed to complementary DNA (cDNA) using a two-step reaction. Total RNA was quantified using a Nanodrop spectrophotometer (Thermo Scientific, DE, USA) and quality assessed using an Agilent 2100 Bioanalyzer (Agilent Technologies, CA, USA). Microarray experiments were performed according to MIAME guidelines. Total RNA from single tumor samples were hybridised to 48K Illumina HumanHT-12 V3.0 Expression BeadChips, covering >25,000 annotated genes from the RefSeq (Build 36.2) and Unigene databases. Arrays were scanned using an Illumina Bead Array™ scanner (Illumina, CA, USA) and probe quality assessed using proprietary software (GenomeStudio, CA, USA). Feature extraction was performed using the Gene Expression Module of the Bead Studio software (Genome Studio, Illumina, Hayward, CA). Data was normalised to the 75^th^ percentile using GeneSpring software (Agilent Technologies). Expression data has been deposited in the National Centre for Biotechnology Information (NCBI) Gene Expression Omnibus (GEO Accession GSE23822).

### Technical validation

Messenger RNA expression levels obtained from microarray analysis in the training set (N = 56), were validated against those obtained using an independent method qRT-PCR. RNA was reverse transcribed to cDNA using a combination of random hexamers (100 ng/µL) and oligo (dT)_15_ primers (200 ng/µL) (Promega, NSW, Australia) in a reaction with 10 mM dNTPs. Pre-validated gene expression assays for candidate genes were purchased from QIAGEN and assayed using SYBR green chemistry (QIAGEN, MD, USA). Five housekeeping genes were co-amplified, and the geometric mean of the housekeepers' expression across individual samples was used for relative quantification. All samples were run in triplicate on an Applied Biosystems 7900HT Fast Machine (Applied Biosystems, Warrington, United Kingdom). Genes were considered technically replicated if the direction of differential expression for candidates was consistent with that from array data, and the magnitude of change was at least 1.2-fold.

### Biological validation

Biological validation was performed on two independent test sets: (1) an independent TPCH set of 12 primary SCC with matching normal lung from our tumor bank, derived from patients whose asbestos exposure status was determined as outlined above, and (2) an independent external dataset of 28 lung cancer samples of various histologies from patients whose asbestos exposure had been assessed by occupational history and lung asbestos fiber counts (Wikman et al [5], [9]). Microarray data was kindly provided by the authors.

### Identification of a prediction panel capable of distinguishing ARLC-SCC/NARLC-SCC

Class prediction analyses were performed in BRB Array Tools using a random variance model and a grid of significance level thresholds (0.01, 0.005, 0.001, and 0.0005) as tuning parameters for classifier development (developed by Dr Richard Simon and Amy Peng Lam, http://linus.nci.hih.gov/~brb/tool.htm). Six different prediction models were used; (1) Compound covariate predictor, (2) Diagonal linear discriminant analysis, (3) K-nearest neighbour, (4) Nearest centroid, (5) Support vector machines and (6) Bayesian compound covariate predictor. The misclassification rate was determined using a leave-one out cross-validation (LOOCV) procedure. With LOOCV, one sample (test) is left out and a classifier developed on the remaining samples. This process is repeated until all samples have been omitted once. A permutation *P*-value for the cross-validated misclassification rate was computed by repeating the cross validation procedure for 1000 permutations of the class label.

### Immunohistochemistry

Immunohistochemical staining was performed using a monoclonal murine anti-human B cell CD20cyIG2akappa antibody targeted to human *MS4A1* (Dako, Denmark, Catalogue Number M0755). Briefly, 4 um sections of formalin-fixed paraffin-embedded tissue were cut onto Menzel Superfrost Plus slides and air dried overnight at room temperature. Sections were dewaxed and rehydrated through a series of xylol and ethanol rinses using a Sakura DRS autostainer. Heat-activated antigen retrieval was performed using the Dako antigen retrieval solution (pH 6.0) and a Biocare Medical Decloaking chamber. The monoclonal primary *MS4A1* antibody was applied at a dilution of 1∶70 and sections counterstained using Mayer's haematoxylin.

### Statistical Analysis

Statistical analyses for microarray experiments were performed using BRB ArrayTools Version 3.8; developed by Dr Richard Simon and Amy Peng Lam, http://linus.nci.hih.gov/~brb/tool.htm. Candidate genes were selected on (a) statistical significance of expression (*P*<0.001) and (b) magnitude of expression change (fold change >2.0). Selection of genes at *P*<0.001 (by Student *t*-test) allowed control of the false discovery rate, limiting the likelihood of selecting false positives. Group comparisons, correlations and associations were performed using χ^2^ tests and two-tailed *t*-tests where appropriate using SPSS statistical software (Version 17, SPSS Inc Chicago, IL, USA). Two tailed *p*-values less than 0.05 were considered statistically significant.

## Results

Summary distributions for age, gender, smoking history and tumor stage for the fifty-six subjects in the training set and twelve test set subjects are shown in Table 1. There were no statistically significant differences in age, gender, smoking history or tumor stage between ARLC-SCC and NARLC-SCC in either the training or test sets (Table 1).

### Class comparison to identify ARLC-SCC associated oncogenes and tumour suppressor genes

**Figure 1:**
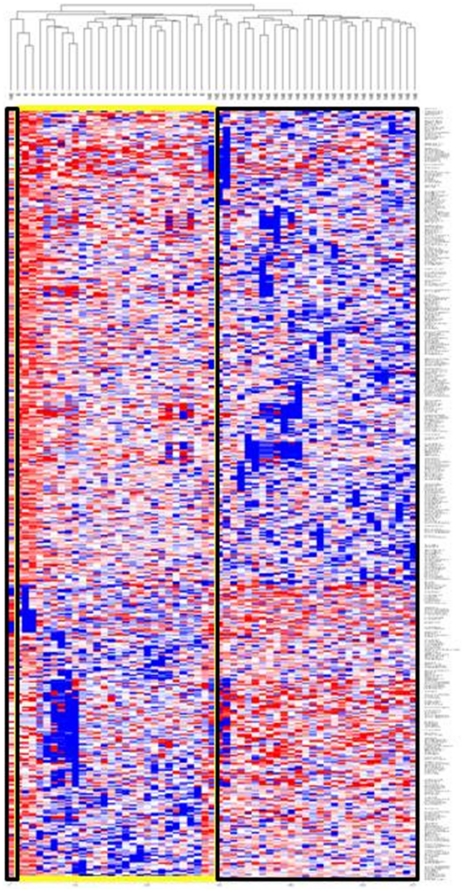
Heat map depicting the genomic profiles of asbestos-related (yellow) and non-asbestos related (black) lung squamous cell carcinomas. Genes were selected on the basis of statistical significance (P<0.05). Genes are represented on the Y axis and tumor samples represented on the X axis. Blue areas represent genes that are under-expressed in the tumor sample while red areas represent genes over expressed in the tumor sample.

Based on a univariate parametric *t*-test, supervised analysis of a total of 8,239 probes in the 56 lung cancers in the training set identified 484 probes differentially expressed between ARLC-SCC (n = 26) and NARLC-SCC (n = 30) (*P*<0.05) with 412 expected by chance. Supervised hierarchical clustering using centred correlation with average linkage showed that these 484 probes were able to distinguish between ARLC-SCC and NARLC-SCC (Figure 1).

**Figure 2:**
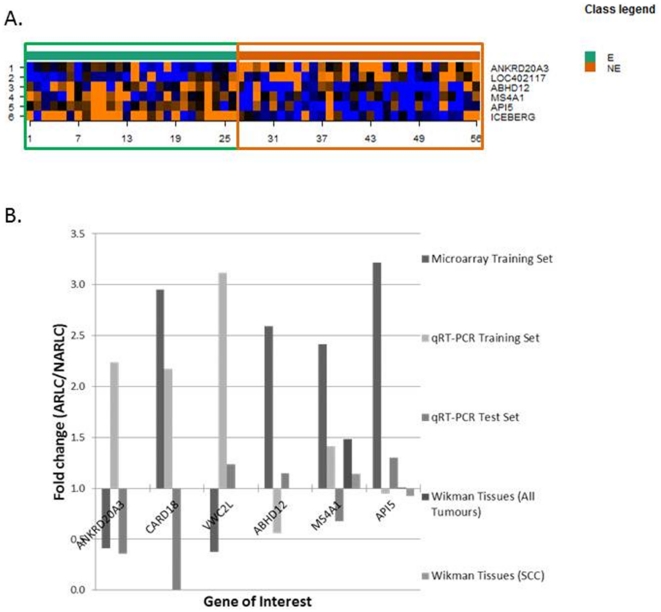
Gene expression differences for the top 6 candidates as measured by microarray and qRT-PCR in the training and test sets. *A. Heat map depicting the genomic profiles of asbestos-related (green) and non-asbestos related (orange) lung squamous cell carcinomas*. Genes were selected on the basis of statistical significance (*P*<0.001) and magnitude of gene expression change (>2.0-fold). Genes are represented on the Y axis and tumor samples represented on the X axis. Blue areas represent genes that are under-expressed in the tumor sample while red areas represent genes over expressed in the tumor sample. *B. Bar graph depicting gene expression changes in TPCH Microarray data, TPCH qRT-PCR (Training and Testing sets) and Wikman Microarray Data*. Fold changes are the mean of ARLC-SCC/mean NARLC-SCC group. TPCH – The Prince Charles Hospital.

To identify individual gene expression markers of ARLC-SCC, we prioritised the 484 genes on the basis of p-value and magnitude of change. Six of these genes satisfied threshold criteria consisting of *P* value<0.001 and >2 fold mean differential expression between groups in microarray analysis (Table 2, Figure 2A). In the same samples, only two genes were independently confirmed as differentially expressed in the same direction as the microarray analysis by the independent method of qRT-PCR: caspase recruitment domain family, member 18 (*CARD18*) and membrane-spanning 4-domains, subfamily A, member 1 (*MS4A1*) (Figure 2B). For both genes the magnitude of higher expression in ARLC compared with NARLC by qRT-PCR was relatively small (<2.5 fold; Table S1).

**Table 2:** Microarray identified differentially expressed genes between ARLC and NARLC in the training set.

Illumina Probe ID	Genbank Accession	Gene Symbol	Gene Description	Fold Change	Chromosome	Map	P-value
**4280288**	NM_021571.2	CARD18	caspase recruitment domain family, member 18	2.894	11	11q21-q22	2.64E-05
**2710615**	NM_015600.3	ABHD12	abhydrolase domain containing 12	2.576	20	20p11.21	3.19E-04
**7050523**	NM_006595.2	API5	apoptosis inhibitor 5	3.204	11	11p12	3.86E-04
**4120669**	NM_001080500.1	LOC402117	von Willebrand factor C domain-containing protein 2-like	−2.664	2	2q34-q35	5.52E-04
**1940010**	NM_152866.2	MS4A1	membrane-spanning 4-domains, subfamily A, member 1	2.371	11	11q12.2	7.42E-04
**4180609**	NM_001012419.1	ANKRD20A3	ankyrin repeat domain 20 family, member A3	−2.400	9	9q12	7.68E-04

The top 6 genes were selected on the basis of high magnitude (>2.0 fold) and statistical significance (p<0.001). These candidates were then biologically validated in independent test sets and technically replicated using an independent method, qRT-PCR.

In our independent test set of 12 primary lung SCC cases *MS4A1* expression was only 1.7 fold higher in tumor tissue (lung SCC) compared with autologous normal lung, whereas *CARD18* was 73 fold higher in tumor (ratio of lung cancer versus normal lung mean; Table S1). This large change is likely due to the small sample size and may not necessarily reflect the normal changes seen in normal lung and lung cancer.

To determine whether the differential expression of these two genes was specific to asbestos related SCC, we interrogated our previous microarray analysis of asbestos related lung adenocarcinoma (ARLC-AC; GSE20875). *MS4A1*, but not *CARD18*, was up regulated in ARLC-AC compared with non ARLC-AC, but the magnitude of fold change was small (FC 1.2, *P* = 0.021; Table S2).

In the external independent validation set of Wikman and co-workers, *MS4A1* was represented by two probes on the Affymetrix HG-U133A microarray chip, used to study gene expression in lung cancers from patients with and without asbestos exposure [5]. Both probes were up-regulated in asbestos related tumours (Probe 1 FC = 1.59; Probe 2 FC = 1.38, Figure 2B; Table S2) but the magnitude of up regulation was again small. *CARD18* was not represented on the Affymetrix HG-U133A chip.

**Figure 3:**
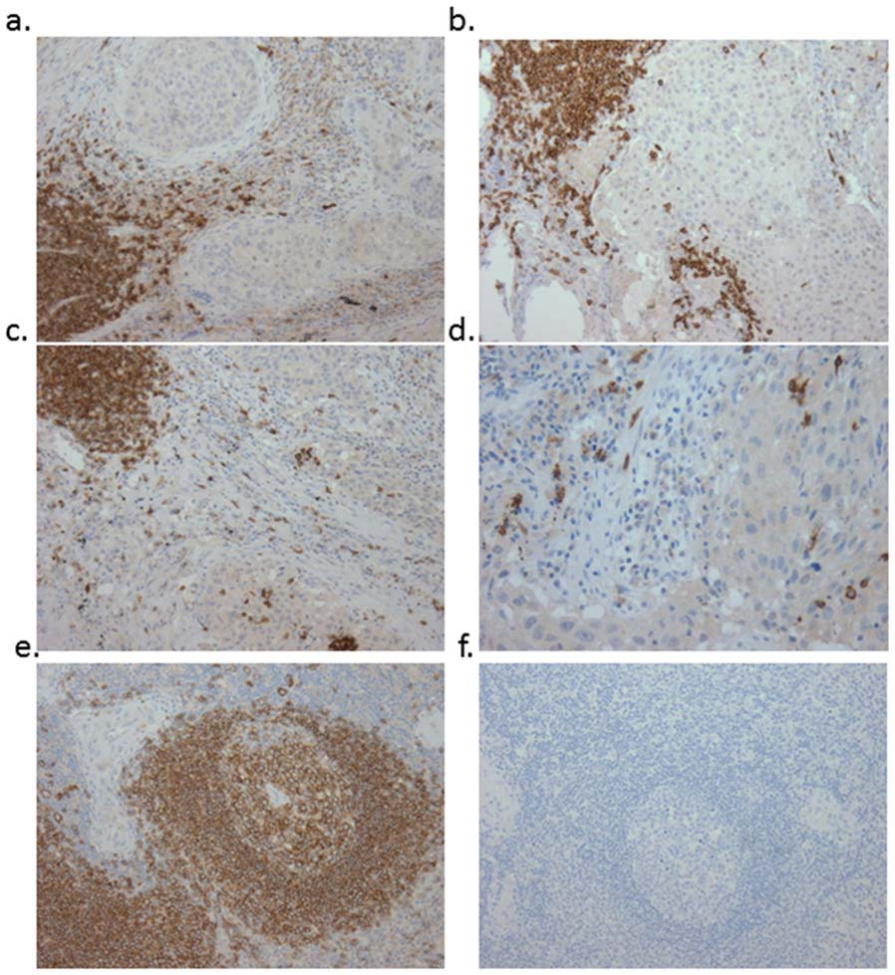
Representative CD20 immunohistochemistry in non-small cell lung cancer at 100×magnification. a and b. Tumor cells fail to stain; positivity seen only in stromal B-lymphocytes. c and d. Tumor cells again negative; CD20-positive B-cells infiltrating tumor nests and stroma. e. Positive control (reactive lymph node). f. Negative control (lymph node without primary antibody).

Because *MS4A1* encodes the CD20 B lymphocyte marker, the origin of its up regulation in ARLC-SCC which comprises primary tumor cells as well as stroma was explored using immunohistochemistry to determine if deregulation was due to stromal lymphocytes rather than lung tumor cells. Fifty-two cases with formalin-fixed paraffin-embedded tumor tissue available for immunohistochemistry were studied with anti-CD20, and no staining was observed in tumor cells. As predicted, the source of *MS4A1* immunopositivity in most cases was stromal lymphocytes. Stromal lymphocytes are often found within the connective tissue septa between nests of tumour cells, and are sometimes accompanied by plasma cells. However CD20 positive tumor infiltrating lymphocytes (TILs) were also observed in seven tumors (six asbestos related, one non-asbestos related). TILs are lymphocytes actually within tumor cell nests, seen in sections as being between or on top of tumor cells. Representative CD20 stains are shown in Figure 3.

### Identification of gene expression profiles to classify ARLC-SCC from NARLC-SCC

Next in order to identify a gene expression classifier capable of distinguishing ARLC-SCC from NARLC-SCC we performed class prediction analysis using six standard models; (1) Compound Covariate, (2) Diagonal Discriminant Analysis, (3) K-nearest neighbor (K = 1,3), (4) Nearest Centroid, (5) Support Vector Machines and (6) Bayesian Compound covariate models. None of these models were able to correctly classify more than 57% of SCCs. The best predictor, K-nearest neighbor (k = 1) misclassified fifteen of twenty-six ARLC-SCC tumors and demonstrated poor test characteristics: sensitivity 42.3%, specificity 70%, positive predictive value 55%, negative predictive value 58.3% (permutation p-value = 0.296 based on 1000 permutations of the class label).

## Discussion

The exact contribution of asbestos to lung cancer burden in modern times is difficult to ascertain with certainty. While asbestos has been shown to chemically and physically interact with DNA, its precise carcinogenic mechanisms in lung and pleura remain unknown. In an attempt to better understand the molecular mechanisms of asbestos related carcinogenicity in squamous cell carcinoma of the lung, we examined the gene expression profiles of 56 SCC; 26 ARLC-SCC (≥20AB/gww) and 30 NARLC-SCC (0 AB/gww) using Illumina gene expression microarrays.

To date, only one study has investigated gene expression profile differences between primary lung cancers from asbestos-exposed individuals versus those without evidence of asbestos exposure. Wikman et al. identified a 47-gene signature capable of distinguishing ARLC/NARLC classes [13], demonstrating the potential to use gene expression profiling to identify asbestos-related tumor biomarkers. We also previously found evidence that gene expression profiles differ between asbestos-related lung adenocarcinoma (ARLC-AC) and adenocarcinoma unrelated to asbestos (NARLC-AC) [9]. Based on these two studies we expected that gene expression profiles would differ between squamous cell carcinomas related and unrelated to asbestos (ARLC-SCC and NARLC-SCC respectively). Our results do not support this hypothesis in that Illumina microarray gene expression profiles were unable to differentiate SCCs according to lung asbestos body counts with enough sensitivity or specificity to be useful as a diagnostic test.

Despite the fact that the Illumina platform has been shown to be highly correlated with Taqman technology [14], only two of the top six differentially expressed genes selected on *p*-value and magnitude of expression difference (*MS4A1* and *CARD18*) were verified by the independent method of qRT-PCR. The limited dynamic range and lower sensitivity of microarrays for gene expression may account for the different results obtained with the more sensitive qRT-PCR method. Secondly, different primer positions relative to the microarray probe locations could reduce consistency between the two methods e.g. if qRT-PCR primers are located far from the microarray probe, correlation may be weaker [15]. We used validated primers from QIAGEN for qRT-PCR and found that they were located close to the microarray probe for 3/6 genes. Although we tried to select primers targeted to the specific transcript the array probe was designed to amplify, undocumented splice variants in some genes could allow preferential amplification of specific transcripts. Several groups have investigated the relationship between microarray and qRT-PCR data reporting higher correlations for up-regulated genes, genes with high magnitudes of change, and genes with lower *p*-values and earlier C_t_ values [16]. Several studies reported lower correlations for genes exhibiting small expression differences (<2-fold) [17], [18]. However, the candidate genes we studied demonstrated relatively high differential expression (>2-fold), making it unlikely that magnitude of expression difference would account for inability to technically validate.

Despite class comparison identifying 484 probes capable of separating ARLC-SCC and NARLC-SCC, there were only two validated genes emerging as potential candidate markers of ARLC-SCC from supervised class comparison analysis. Despite selecting candidates at stringent *P*-values (*P* = 0.001) and high magnitude (>2-fold change), probes contained within the original 484-probe profile exhibited very high false discovery rates. Nonetheless, the study was sufficiently powered to detect differences between the two groups. This may reflect lack of biological differences between ARLC-SCC and NARLC-SCC.

Not surprisingly then, class prediction analysis failed to identify a primary tumor signature capable of distinguishing ARLC-SCC and NARLC-SCC with adequate sensitivity and specificity to be useful as a clinical test. This also suggests biological similarity between ARLC and NARLC.

The two qRT-PCR validated candidate genes from microarray class comparison, *MS4A1* and *CARD18* were identified as up regulated in ARLC-SCC compared with NARLC-SCC. Although expression of *CARD18* was markedly elevated in tumor tissue of squamous cell lung cancers compared with that of autologous non-tumor lung tissue, the magnitude of differential expression between ARLC-SCC and NARLC-SCC was modest. This gene was not differentially expressed in lung adenocarcinomas according to asbestos exposure. Taken together, these observations are consistent with high expression of *CARD18* in SCC, but suggest that *CARD18* is not a good discriminatory marker for ARLC-SCC versus NARLC-SCC.

The protein product of *CARD18*, ICEBERG, is a 90 amino acid protein encoded on 11q22.3, and a member of the death domain superfamily whose members form protein-protein interactions (homodynes or heterodimers) to function as adaptors in signaling pathways or to recruit other proteins into signaling complexes. ICEBERG is a CARD (capsize activation and recruitment domain) only protein, induced by pro-inflammatory stimuli. It binds to the prodomain of caspase-1 preventing its association with RIP2 and inhibiting synthesis of interleukin-1-beta. While some CARD only proteins activate the nuclear transcription factor NF-κB, there is no evidence that ICEBERG is involved in its activation [19], so the significance of its elevated expression in lung SCC and particularly asbestos related SCC is unknown. As our tumor samples were not micro-dissected or otherwise enriched for tumor content, it is unknown whether the *CARD18* signal in squamous cell carcinoma originates from tumor cells themselves or from stromal elements.

The other gene with verified differential expression between ARLC-SCC and NARLC-SCC was *MS4A1*. Its level of expression was somewhat higher in SCC tumor tissue compared with non-malignant autologous lung, and in ARLC (both SCC and AC) compared with NARLC. Although of small magnitude, the differential expression of this gene was consistent and was associated with ARLC of both major histopathologic subtypes. *MS4A1* expression has also been shown to be inducible by crocidolite in *in vitro* model systems of asbestos exposure.

The *MS4A1* gene located on 11q12 encodes a member of the membrane-spanning 4-domains, subfamily A, B lymphocyte antigen CD20, which plays a role in differentiation of B lymphocytes into plasma cells [20]. Inhibitory monoclonal antibodies to CD20 such as rituximab are therapeutic in several hematologic malignancies and immunologically driven inflammatory diseases including rheumatoid arthritis [21]. Apart from its classical expression in B-lineage haemopoetic cells, CD20 has been associated with aggressive clinical behavior in a microarray gene expression study of melanoma [22]. Intriguingly tumorigenic melanoma cells were found to be enriched within a CD20+ fraction, suggesting that melanoma cancer stem cells are pluripotent and express CD20 [23]. If the CD20 signal in ARLC originated in pluripotent lung cancer stem cells, the enticing possibility of targeting these specifically with existing monoclonal antibody therapeutics arises. To explore this possibility and determine the cellular source of CD20 within lung cancers, we studied the localization of CD20 protein in fifty-two SCCs using immunohistochemistry with anti-CD20 antibodies. The CD20 signal appeared to be primarily of lymphocytic rather than tumor cell origin, with the majority of the signal arising from stromal lymphocytes. A small proportion of tumors also showed CD20 staining in tumor infiltrating lymphocytes (TILs). Nevertheless, the absence of tumor cell CD20 staining in all fifty-two cases and positive stromal lymphocyte immunostaining in most tumors indicates that the CD20 expression signal observed in microarray and qRT-PCR almost certainly originates from lymphocytes located mainly within stroma.

In our microarray expression analysis of macro-dissected tumors, the finding of a gene of interest predominantly expressed in stromal rather than tumor cells raises a significant issue concerning the method of tumor sample preparation, and indicates the possibility of a stromal effect from asbestos. The effect of the proportion of stromal content on results of tumor gene expression profiling has been reported. In some studies the method of dissection (macro versus micro) was found to have only minor effects on the tumor gene expression profile [24], [25]. Conversely, Klee and colleagues showed that laser micro dissection (LCM) was capable of identifying differentially expressed genes not identified using bulk dissection methods [26] suggesting that LCM provides greater sensitivity for detection of differentially expressed markers. While debate continues, LCM based sample preparation would seem to be preferred where the goal is to identify tumor specific markers as it provides tumor cell enrichment.

There is increasing recognition that cancer cells rely on the surrounding microenvironment to driver the cancer phenotype favouring survival, growth and spread [27]. Tumor behaviours such as progression and prognosis are dependent on cellular interactions between tumor cells and stromal elements including immune cells and cells of mesenchymal origin [27]. For example ‘reactive stroma’ containing fibroblasts and myofibroblasts characterise numerous invasive cancers including lung cancer with bi-directional cross-talk between tumor cells and stromal elements important for fibroblast differentiation [27]. In particular myofibroblasts and fibroblasts provide an important source of extracellular matrix proteins which are important for development of the extracellular matrix in tumor stroma [27]. Inclusion of stromal elements in study samples is therefore important, enabling discovery of potentially important information about the tumor microenvironment. Thus, the finding of a gene expressed predominantly in the stroma alludes to the possibility of a tumor-stroma interaction generated by asbestos.

A potential limitation of this study is the method of sample preparation used. As the primary aims were to identify gene expression profile differences and differentially expressed genes with potential diagnostic or therapeutic relevance, if redesigning this study we would use microdissected samples. Our results are of interest in highlighting the critical importance of methodology to interpretation of results in high dimensional gene expression studies, and of the need for verification with independent methods. Future gene expression studies should concentrate on determining their study aims and relevant methodology before embarking on microarray profiling experiments to ensure their question is answered adequately.

In summary, in this study we identified gene expression profile differences between ARLC-SCC and NARLC-SCC, but failed to verify the differential expression of individual genes that were highly significant in microarray analysis using the independent method of qRT-PCR. We found *CARD18*, encoding the capsize 1 interacting protein ICEBERG, specifically up-regulated but in small magnitude in ARLC-SCC (but not ARLC-AC). We also found *MS4A1* encoding the B-lymphocyte marker CD20, to be up-regulated in ARLC (both SCC and AC), and traced this signal to stromal lymphocytes rather than tumor cells.

The finding of a candidate gene primarily expressed in stromal lymphocytes rather than tumor cells suggests that study design and sample preparation methods must be considered when interpreting microarray study results. This study also calls for more research to determine the possible role of *MS4A1* in ARLC given it was found in both AC and SCC. For our purposes, tumor cell enrichment by microdissection may have avoided the emergence of dominant signals from stromal elements, as illustrated by identification of *MS4A1* as a gene of interest. Conversely, it also demonstrates the advantage of using macrodissection by allowing an appreciation of the contribution of stroma which is known to be important in cancer development [27]. On the other hand, microdisection for gene expression studies enables more precise identification of gene dysregulation in lung cancer cells only; similarly for clonal cell lines. Our findings however suggest both of these study designs are helpful and indeed complementary, and stress the need for independent validation and replication Future studies attempting to identify expression markers of asbestos related SCC should carefully consider this and other design aspects to achieve their specific goals.

## Supporting Information

Table S1 **Gene Expression values of candidate genes in TPCH Microarray data, TPCH qRT-PCR (Training and Test Sets) and Wikman Microarray Data.**Fold Changes are mean ARLC/mean NARLC. Where there was multiple probes for the gene, the mean expression across all array probes was used. *Average fold change of all probes used.
(XLSX)

Table S2 **Gene expression values for the Wikman and Wright Lung Adenocarcinoma Microarray Data.** Fold changes for each probe on the array are tabulated. Fold changes for each probe on the array are tabulated.
(XLSX)

## References

[1] Surveillance Epidemiology and End Results (SEER) Program SEER*Stat Database: Incidence - SEER 9 Regs Public-Use, Nov 2004 Sub (1973-2002)

[2] Peto R, Darby S, Deo H, Silcocks P, Whitley E (2000). Smoking, smoking cessation, and lung cancer in the UK since 1950: combination of national statistics with two case-control studies.. Bmj.

[3] Sun S, Schiller JH, Gazdar AF (2007). Lung cancer in never smokers - a different disease.. Nat Rev Cancer.

[4] Henderson DW, Jones ML, De Klerk N, Leigh J, Musk AW (2004). The diagnosis and attribution of asbestos-related diseases in an Australian context: report of the Adelaide Workshop on Asbestos-Related Diseases. October 6–7, 2000.. International Journal of Occupational & Environmental Health.

[5] Wikman H, Ruosaari S, Nymark P, Sarhadi VK, Saharinen J (2007). Gene expression and copy number profiling suggests the importance of allelic imbalance in 19p asbestos-associated lung cancer.. Oncogene.

[6] Lee BW, Wain JC, Kelsey KT, Wiencke JK, Christiani DC (1998). Association of cigarette smoking and asbestos exposure with location and histology of lung cancer.. Am J Respir Crit Care Med.

[7] Roggli VL, Sanders LL (2000). Asbestos content of lung tissue and carcinoma of the lung: a clinicopathologic correlation and mineral fiber analysis of 234 cases.. The Annals of occupational hygiene.

[8] de Klerk NH, Musk AW, Eccles JL, Hansen J, Hobbs MS (1996). Exposure to crocidolite and the incidence of different histological types of lung cancer.. Occup Environ Med.

[9] Wright CM, Larsen JE, Hayward NK, Martins MU, Tan ME (2010). ADAM28: a potential oncogene involved in asbestos-related lung adenocarcinomas.. Genes Chromosomes Cancer.

[10] Roggli VL, Roggli VL, Greenberg SD, Pratt PC (1992). Appendix - Tissue Digestion Techniques in Pathology of Asbestos-Associated Diseases..

[11] Wright CM, Bowman RV, Tan ME, Martins MU, McLachlan RE (2008). Lung asbestos content in lungs resected for primary lung cancer.. J Thorac Oncol.

[12] Schneider F, Sporn TA, Roggli VL (2008). Crocidolite and mesothelioma.. Ultrastructural pathology.

[13] Wikman H, Ruosaari S, Nymark P, Sarhadi VK, Saharinen J (2007). Gene expression and copy number profiling suggests the importance of allelic imbalance in 19p in asbestos-associated lung cancer.. Oncogene.

[14] Shi L, Reid LH, Jones WD, Shippy R, Warrington JA (2006). The MicroArray Quality Control (MAQC) project shows inter- and intraplatform reproducibility of gene expression measurements.. Nature biotechnology.

[15] Etienne W, Meyer MH, Peppers J, Meyer RA (2004). Comparison of mRNA gene expression by RT-PCR and DNA microarray.. Biotechniques.

[16] Morey JS, Ryan JC, Van DFM (2006). Microarray validation: factors influencing correlation between oligonucleotide microarrays and real-time PCR.. Biological procedures online.

[17] Rajeevan MS, Vernon SD, Taysavang N, Unger ER (2001). Validation of array-based gene expression profiles by real-time (kinetic) RT-PCR.. The Journal of molecular diagnostics : JMD.

[18] Wurmbach E, Yuen T, Sealfon SC (2003). Focused microarray analysis.. Methods (San Diego, Calif.

[19] Druilhe A, Srinivasula SM, Razmara M, Ahmad M, Alnemri ES (2001). Regulation of IL-1beta generation by Pseudo-ICE and ICEBERG, two dominant negative caspase recruitment domain proteins.. Cell death and differentiation.

[20] Tam CS, Otero-Palacios J, Abruzzo LV, Jorgensen JL, Ferrajoli A (2008). Chronic lymphocytic leukaemia CD20 expression is dependent on the genetic subtype: a study of quantitative flow cytometry and fluorescent in-situ hybridization in 510 patients.. Br J Haematol.

[21] Sacchi S, Federico M, Dastoli G, Fiorani C, Vinci G (2001). Treatment of B-cell non-Hodgkin's lymphoma with anti CD 20 monoclonal antibody Rituximab.. Critical reviews in oncology/hematology.

[22] Bittner M, Meltzer P, Chen Y, Jiang Y, Seftor E (2000). Molecular classification of cutaneous malignant melanoma by gene expression profiling.. Nature.

[23] Fang D, Nguyen TK, Leishear K, Finko R, Kulp AN (2005). A tumorigenic subpopulation with stem cell properties in melanomas.. Cancer research.

[24] de Bruin EC, van de Pas S, Lips EH, van Eijk R, van der Zee MM (2005). Macrodissection versus microdissection of rectal carcinoma: minor influence of stroma cells to tumor cell gene expression profiles.. BMC Genomics.

[25] El-Serag HB, Nurgalieva ZZ, Mistretta TA, Finegold MJ, Souza R (2009). Gene expression in Barrett's esophagus: laser capture versus whole tissue.. Scand J Gastroenterol.

[26] Klee EW, Erdogan S, Tillmans L, Kosari F, Sun Z (2009). Impact of sample acquisition and linear amplification on gene expression profiling of lung adenocarcinoma: laser capture micro-dissection cell-sampling versus bulk tissue-sampling.. BMC Med Genomics.

[27] Hiscox S, Barrett-Lee P, Nicholson RI (2011). Therapeutic targeting of tumor-stroma interactions.. Expert opinion on therapeutic targets.

